# Supplementary material for: Advancing urban management: Integrating GIS, LLMs, and media narratives into environmental and socio-economic analyses for enhanced urban crime analysis
Source: PLoS One. 2025 Nov 26;20(11):e0331788. doi: 10.1371/journal.pone.0331788 (PMC12654902; doi:10.1371/journal.pone.0331788)
Supplement: Attachment — Submitted filename: Response to Reviewers.docx [file pone.0331788.s002.docx]

**Reviewer #1:**

**- Comments: There are some typo errors. For example, see Fig. 5. The local Moreland index. I think you mean 'Local Moran's I'.**

- Thank you for pointing this out. We have corrected the typo errors, including the reference to the 'Local Moran's I' in Figure 5. Additionally, we carefully reviewed the manuscript to address any remaining language issues, ensuring improved clarity and readability.

**- 2-Please upload high-quality images and maps in the manuscript. Numerous figures in the file that was uploaded cannot be read.**

- Thank you for your feedback. We have re-uploaded the high-resolution versions of all images to ensure clarity and readability.

**- 3-Where and how large a language model was used? I did not see anything in the methodology section or results. The situation is unclear.**

- We have implemented few-shot learning methods for text classification of media-reported crimes and provided a detailed explanation in the newly renamed “Data and Methods” section. Specifically, subsection 2.2 is now titled “Use of LLM in Media Reporting” and includes comprehensive details on applying LLMs in our study. The classification results are utilized to compare media-reported data with actual crime data in section 3.1, “Comparison Between Media Data and Crime Data,” enhancing the clarity and depth of our analysis.

**- 4- I think the discussion section is weak. It is very useful to mention your findings, compare them with previous study findings, and discuss them. For example, I think several studies have been conducted about crimes in Chicago in recent years.**

- Thank you for your valuable suggestion. We have incorporated references to recent studies on the impact of Chicago's environment on crime, as well as research on how media-reported crimes influence public perception. These references are now included in the discussion section to connect our findings with existing literature, thereby providing a more comprehensive context for our results.

**Reviewer #2:**

**Originality**

**- The integration of GIS, LLMs, and media narratives is a novel approach in the field of urban crime analysis. The study stands out by addressing the interplay between environmental, socioeconomic factors, and media reporting, which provides a fresh perspective compared to traditional crime studies. The originality is clear, and I was unable to find significant overlap in other published papers.**

- Thank you for your feedback! We are glad that you appreciate our work.

**Abstract**

**- The abstract effectively summarizes the research objectives, methodology, and findings. However, the term "GIS" is used without prior explanation. I recommend that the authors introduce "Geographic Information System" before using the acronym, ensuring clarity for readers unfamiliar with the term. Other than this, the abstract provides a concise overview of the study’s key points.**

- Thank you for your feedback. We have addressed this issue.

**Introduction**

**- The introduction offers a detailed and well-researched background on the relationship between media reporting and crime perception. It successfully sets up the study’s objectives, but Section 1.1 is somewhat verbose and could be condensed into a single paragraph. Streamlining this part will improve readability while retaining the critical points. Additionally, when discussing correlations between factors such as temperature and crime, the distinction between correlation and causation should be more clearly stated to avoid any potential misinterpretation.**

- Thank you for pointing this out. We have substantially rewritten Section 1.1 to improve readability by making it more concise while keeping the original information as much as possible. We also add contents to distinguish the correlation and causation in the Introduction part.

**Methodology**

**- The methodology is one of the manuscript's strongest sections, providing a clear and structured approach. The use of GIS crime data, combined with media reporting analysis using GPT-3.5, is well-executed. The authors’ innovative application of LLMs to classify crime reports with high accuracy is commendable, and the spatial analysis using Geographically Weighted Regression (GWR) is solidly grounded in spatial econometrics. Overall, this section demonstrates rigorous technical standards.**

- Thank you for your appreciation.

**Results**

**- The results section is comprehensive and provides clear insights into the discrepancies between media-reported and actual crime data. The authors effectively highlight key findings, such as the media’s overrepresentation of certain crimes (e.g., homicide) and underreporting of others (e.g., battery). However, distinguishing between different levels of theft (e.g., petty vs. grand theft) would offer more nuance to the analysis of surveillance impact.**

- Thank you for pointing out the issue. We have corrected the section title from "Results" to "Discussion."

**Discussion**

**- The discussion is well-structured, synthesizing the results with broader literature. The spatial analysis findings are plausible and align well with the study’s hypotheses. However, the policy recommendations, while relevant, could be more specific. The suggestions currently offered are somewhat general and would benefit from more targeted advice based on the study’s spatial findings. Providing more actionable insights would enhance the practical application of the study’s conclusions for urban planners and policymakers..**

- Thank you for pointing this out. We agree that the policy recommendations could be more specific and actionable. We had revised this section to include more detailed, targeted advice.

**Data and Code Availability**

**The transparency in making the environmental, socioeconomic, and media data available through publicly accessible sources like the Chicago Data Portal and Perigon API is commendable. The authors’ adherence to open science practices ensures that the study can be replicated, which is a strength of the manuscript.**

- Thank you for your appreciation.

**Recommendation**

**The manuscript presents a novel and well-executed study that integrates multiple analytical tools to examine urban crime dynamics. The study is methodologically sound and contributes valuable insights to both academic and practical discussions on urban management. However, I recommend minor revisions to improve clarity and expand on certain sections, particularly in explaining correlations, streamlining the introduction, and enhancing the specificity of policy recommendations. Once these revisions are made, the paper will be well-suited for publication in PLOS ONE.**

- Thank you for your constructive feedback. We have revised the manuscript to enhance the explanation of the correlations and streamlined the introduction for better clarity. Additionally, we have expanded the specificity of our policy recommendations, particularly for different community groups in the Chicago area, including suggestions from media and community stakeholders.
