# Supplementary material for: Advancing urban management: Integrating GIS, LLMs, and media narratives into environmental and socio-economic analyses for enhanced urban crime analysis
Source: PLoS One. 2025 Nov 26;20(11):e0331788. doi: 10.1371/journal.pone.0331788 (PMC12654902; doi:10.1371/journal.pone.0331788)
Supplement: Attachment — Submitted filename: to_reviewer.docx [file pone.0331788.s003.docx]

Response to Reviewer 1 Comments

**Point 1.1:**

Reviewer #1: I am pleased to confirm that the authors have successfully addressed all the comments and suggestions I previously provided.

**Response 1.1:** **Thank you for your recognition.**

Response to Reviewer 2 Comments

**Point 2.1:**

Reviewer #2: The authors have addressed all the comments I raised in the previous review. The introduction of GIS is now clearer, the results include more commentary about different nuances of crimes, and the policy recommendations are more specific and practical. The manuscript is technically sound, and the methods and data are well-explained. I think the paper is now well-written and ready to be published.

**Response 2.1:** **Thank you for your recognition and for your suggestion in improving this paper.**

Response to Reviewer 3 Comments

Reviewer #3: Advancing Urban Management
The manuscript raises interesting points and explores connections between crime reporting, urban design, and socio-economic variables. I am aware that there has been another round of reviewers before me and their comments have been addressed. However, I have serious concerns regarding the presentation and rationale for methodological choices. I am concerned about how the data is analysed and the interpretation of the results. The policy recommendations must be seriously considered and analyse if this is a result of the research. Below I give the authors some feedback and suggest a redesign of their study and submit the manuscript elsewhere.

**Point 3.1:**

First, regarding the variables.
Regarding the points of Interest, the high heterogeneity in POI counts suggests a potential data issue. The locations with 400+ POIs seems wrong. Or the data has some POI that are less relevant than others.

**Response 3.1:**

Thank you for raising this concern. The POI data in our study were sourced from OpenStreetMap, which includes a wide range of features such as restaurants, retail outlets, public amenities, and service points. Locations with over 400 POIs primarily correspond to downtown Chicago and other commercial hubs where such density is realistic given the concentration of businesses and services. These high POI counts reflect the actual urban heterogeneity rather than data errors. We retained all POIs because our objective was to capture the full scope of territoriality and activity support, as emphasized in CPTED theory. To avoid misinterpretation, we will add a clarification in the Methods section 2.3.1 to indicate that while some POIs may be less directly relevant to crime dynamics, their inclusion ensures consistency and comprehensiveness in measuring environmental complexity.

**Point 3.2:** Population counts of 10,000+ people in a small area? This raise questions about the data's validity. How is there significant population in that location, but without corresponding housing?
**Response 3.2:**

Thank you for mentioning this point. We re-examined the issues with the code and the original data. Previously, the display problem was due to problems with the legend range display of the regional values in the code. We re-output all the visualization results based on the gis software.

**Point 3.3:** For the water area, elaborate on its role in the analysis and how it contributes to the study's aesthetic dimension. Some variables, like the southern lake in Chicago shown on the map, appear disproportionately large. Clarify what these variables represent. And what is the rationale. It cannot be that I just push the data into some ML algorithm, and then something comes out so it must be true. Why water? How would this look like in a non-coastal city?

**Response 3.3:** Thank you for your suggestions and they are indeed valuable. The inclusion of water area is grounded in Crime Prevention through Environmental Design, specifically the Image Maintenance dimension, which relates to environmental aesthetics and quality. Water bodies in urban environments, such as Lake Michigan and riverfront areas in Chicago mentioned in our article, typically are associated with improved walkability, increased recreational activity, and natural surveillance, all of which can influence crime dynamics. Even if these areas may appear disproportionately large on maps, they represent the actual geographic features that shape urban design and usage patterns since Chicago is next to the lake. In non-coastal cities, comparable variables would include reservoirs, riverfronts, or major parks, as the underlying principle is to capture open space aesthetics and their effect on human activity. This is not a purely data-driven inclusion; rather, it reflects how environmental quality impacts crime risk, especially the quality of coastal infrastructure. We will clarify this rationale in the Methods section, at the end of 2.3.1, for generalizability and clarity.

**Point 3.4:**

The rationale of the Greenhouse Gas Emissions as Proxy for activity levels seems incorrect (like a fancy hotel or restaurant has little GHG, but a factory has loads). Clarify whether this is your contribution or based on prior research. If the latter, cite the appropriate source. If original, provide justification for its use in this context.

**Response 3.4:** Thank you for this comment. We have clarified the rationale for using GHG emissions as a proxy for urban economic and activity intensity, referencing prior studies that link emissions with land-use and mobility patterns. We also acknowledge its limitations (e.g., industrial zones vs. commercial areas) and added this discussion, along with a note suggesting future integration of direct mobility measures, to the Methods and Limitations sections.

**Point 3.5:** Second, regarding some methods.
Some terms are introduced without any intuition, reference or context, for example, the Cohen's Kappa. I suggest an intuitive explanation of Cohen's kappa, its relevance to the study, and why a value of 0.92 is considered high. Additionally, include a citation demonstrating its application in this context.

**Response 3.5:** Thank you and this is a helpful suggestion. We’ve added an intuitive explanation of Cohen’s kappa in the Methods section and provided citations to describe its interpretation. We also clarify that our κ = 0.92 falls within the “almost perfect” agreement range.

**Point 3.6:** The same goes with the Perigon Database. A brief explanation of what the Perigon database is, how it is compiled, and its relevance to crime research. Are all news events included in this database? Add a citation to support its use in this field.

**Response 3.6:** Thank you for the suggestion. We clarified the details about Perigon database.

**Point 3.7:** Categorization of Crimes. Why is it necessary to categorize reported crimes into designated categories? What are the "three distinct crime typologies," and who determined these categories? Clearly define and justify the choice of categories with proper references.

**Response 3.7:** Thank you for raising this point. The categorization of reported crimes follows the official classification used by the Chicago Data Portal, which aligns with the Illinois Uniform Crime Reporting (UCR) system. This ensures consistency between media reports and official crime data, enabling comparative analysis. The “three distinct crime typologies” (theft, criminal damage, and battery) were selected based on their prevalence in the Chicago Open Data dataset. We have clarified this in the Methods section 2.2 and added citations to the official data source.

**Point 3.8:** For concepts such as "surveillance" (measured by road network density), clarify whether this metric is based on prior work (with a proper citation) or is a novel contribution. If it is the second, explicitly state that the metric is proposed as a proxy to study surveillance. The same applies to "maintenance" (using the National Walkability Index) and "total water area" (linked to aesthetics).

**Response 3.8:** Thank you for raising these questions. These variables are mainly based on summarizations from previous studies, and we have added citations to corresponding research. Research shows that street connectivity is an important physical environmental feature for understanding criminal opportunities and guardianship, and enhancing road network connectivity and density can increase pedestrian and vehicle activity, boost natural surveillance and reduce theft opportunities. Natural surveillance maximizes the visibility of spaces and their users through the design layout of physical features, activities, and people.

Walkability as an environmental maintenance indicator is based on CPTED's Image/Maintenance principle. The "broken windows theory" emphasizes that "neglected areas invite crime, which reinforces the need for good property maintenance to assert visible ownership of space." Maintenance focuses on keeping areas clean and well-maintained. Places with litter, graffiti, and overgrown landscaping send a signal that no one cares what happens in that area. The use of water area as an environmental quality indicator falls under exploratory research. Although existing studies mainly focus on the health benefits and socio-economic impacts of urban blue spaces, there is a lack of literature directly studying the relationship between water bodies and land crimes. Our analysis is based on theoretical assumptions regarding environmental aesthetics and the potential impact of open spaces on community cohesion.

**Point 3.9:** Regarding the Socio-Economic Characteristics, there is a phrase like "Socio-economic characteristics have been extensively discussed in previous re-search, demonstrating a significant correlation with crime rates." lacks a citation. Well, can you actually say that? (And research is a single word).

**Response 3.9:** Thank you for providing the feedback. We corrected the typo (“research”) and added citations to substantiate the claim that socio-economic characteristics are strongly associated with crime rates in prior studies.

**Point 3.10** Third, regarding the results and conclusions
The policy recommendations are basically good wishes but far from the scope of the manuscript and the results, and with a lack of academic rigour. The findings do not really suggest how to prevent crime, but they present some correlation with media. This is fine. One cannot solve everything in a single paper. However, making policy recommendations is not just adding one last paragraph to your manuscript. Phrases like "limit excessive connectivity" make me feel like the implications were not considered seriously and were added just to say that it has policy implications. Yes, cities could "restrict potential escape routes for offenders", but what budget is needed? What implications in terms of road traffic would this proposal have? Would a bus route be blocked by this idea? Can ambulances or firefighters travel through the city in case of an emergency?

**Response 3.10:** Thank you for this point. We agree that our original policy recommendations lacked sufficient grounding in the study’s findings. We have revised the first two paragraphs in Section 4.3 to ensure recommendations are explicitly tied to the evidence presented in the Results and Discussion sections. Instead of general prescriptions, we now provide nuanced interpretations of how observed spatial and socio-economic correlations might inform planning strategies, while removed prescriptive statements.

**Point 3.11.** The same with "media outlets should...". Media is a business, and as such, its objective is to make money. If only 1/1000 crimes appear in the news, should they start reporting minor thefts over a mass shooting, only to be more accurate?

**Response 3.11:** Thank you for this point. We acknowledge that the suggestions we provided were immature and that the media operates under market-driven incentives, which makes it unrealistic to expect equal coverage of all crime types. Our intention was not to prescribe media practices, but to highlight the societal implications of selective reporting. We have revised this section to remove normative language and instead focus on the theoretical relevance of media narratives shaping public perception and policy. We now frame this as an observation of structural constraints rather than a recommendation for content redistribution.

**Point 3.12.** Minor comments
All equations are punctuated and integrated into the text following proper academic conventions. See https://www2.math.uconn.edu/~khlee/math2784s10/writingtips.pdf
**Response 3.12:**

Thank you for your suggestion. We have adjusted the table and description of the formula based on the standards of previous studies.

**Point 3.13.** The map with a black background is inconsistent with others and difficult to interpret. Revise for consistency and readability.

**Response 3.13:**

Thank you for raising this point. We have modified and unified the background colors of all images and ensured readability.

**Point 3.14.** The results follow a weird narrative. Why start with the number of crimes across months, if there is almost no variation across time? Present results from general to specific for better narrative flow. Start with the total number of crimes reported in the media, then compare media reports to actual crime data, followed by geographical mapping. The time series analysis, while interesting, conveys minimal new insights and might be better placed in supplementary materials.

**Response 3.14:**

Thank you for mentioning this. We have revised the narrative logic at the beginning of section 3.1. Now we start the description from the general conclusion and weaken the results of the time series.
