# Supplementary material for: Advancing urban management: Integrating GIS, LLMs, and media narratives into environmental and socio-economic analyses for enhanced urban crime analysis
Source: PLoS One. 2025 Nov 26;20(11):e0331788. doi: 10.1371/journal.pone.0331788 (PMC12654902; doi:10.1371/journal.pone.0331788)
Supplement: Attachment — Submitted filename: review.docx [file pone.0331788.s004.docx]

Response to Reviewer 3 Comments

Reviewer #3:

The authors modified the manuscript following all my previous suggestions. I only have a few comments for them.
**Point 1.1：**Please check the following citations since they have a typo:
[22] resi-dential
[25] Lan-guage
[29] kohen (is a name, so write "Kohen". Also "sage"
[54] chicago
[60] chicago, covid
[61] shiraz, iran
[62] Belgium
**Response 1.1:**

**Thank you for pointing out these issues. We recheck and rewrite these typo issues.**

**Point 1.2：**After the expression (3), the definition of w_ij does not need the curly brackets.
The (2) for the second expression should be in the same line as the expression.
**Response 1.2: Thank you for mentioning this. We removed the curly brackets, and also fixed the order (2) place issues.**

**Point 1.3：**The section 4.3 is called "Policy Recommendations" but I suggest changing this subtitle. I am happy that you dropped some of the recommendations that you wrote before, but now (better) that there are no punctual recommendations, then the subtitle seems weird. And I would suggest that the whole "Discussions" section could fit on a single page, from the three pages that you have now. Usually, on a manuscript, less text but more impactful is better.

**Response 1.3: Thank you for your suggestion. We had rewritten the whole discussion section. And make these contexts more concise and clearer and keep the impactful thinking for future readers.**
